# *Assessing animal welfare impact of fourteen control and dispatch methods for house mouse (*Mus musculus*), Norway rat (*Rattus norvegicus*) and black rat (*Rattus rattus*)*

**DOI:** 10.1017/awf.2022.2

**Published:** 2023-01-26

**Authors:** Ciska De Ruyver, Kristof Baert, Emma Cartuyvels, Lies AL Beernaert, Frank AM Tuyttens, Herwig Leirs, Christel PH Moons

**Affiliations:** 1 Department of Veterinary and Biosciences, Ethology and Animal Welfare Research Group, Faculty of Veterinary Medicine, Ghent University, Heidestraat 19, 9820 Merelbeke, Belgium; 2 Wildlife Management and Invasive species, Research Institute for Nature and Forest (INBO), Havenlaan 88 bus 73, Brussels, Belgium; 3 Department of Biotechnology, Vives University College, Wilgenstraat 32, 8800 Roeselare, Belgium; 4 Animal Sciences Unit, Flanders Research Institute for Agriculture, Fisheries and Food (ILVO), Scheldeweg 68, 9090 Melle, Belgium; 5 Evolutionary Ecology Group, University of Antwerp, Universiteitsplein 1, 2610 Wilrijk, Belgium

**Keywords:** animal welfare, *Mus musculus*, pest control, *Rattus norvegicus*, *Rattus rattus*, welfare assessment

## Abstract

Population control of the house mouse (*Mus musculus*), Norway rat (*Rattus norvegicus*) and black rat (*Rattus rattus*) is common practice worldwide. Our objective was to assess the impact on animal welfare of lethal and non-lethal control methods, including three dispatch methods. We used the Sharp and Saunders welfare assessment model with eight experts scoring eleven control methods and three dispatch methods used on the three species. We presumed the methods were performed as prescribed, only taking into account the effect on the target animal (and not, for example, on non-target catches). We did not assess population control efficacy of the methods. Methods considered to induce the least suffering to the target animal were captive-bolt traps, electrocution traps and cervical dislocation, while those with the greatest impact were anticoagulants, cholecalciferol and deprivation. Experts indicated considerable uncertainty regarding their evaluation of certain methods, which emphasises the need for further scientific research. In particular, the impact of hydrogen cyanide, chloralose and aluminium phosphide on animal welfare ought to be investigated. The experts also stressed the need to improve Standard Operating Procedures and to incorporate animal welfare assessments in Integrated Pest Management (IPM). The results of our study can help laypeople, professionals, regulatory agencies and legislators making well-informed decisions as to which methods to use when controlling commensal rodents.

## Introduction

The house mouse (*Mus musculus*), Norway rat (*Rattus norvegicus*) and black rat (*Rattus rattus*) are commensal rodents frequently found in urban settings, on farms and in the wild (Castle 1947; Traweger *et al.* 2006; Jones *et al.* 2013; Modlinska & Pisula 2020). Approximately 44% of all mammalian species are rodents, of which 5 to 10% are considered to cause a problem for humans in both rural and urban areas (Panti-May *et al.* 2012). Although there is no precise estimation of population sizes of house mice, Norway and black rats, outbreaks and infestations of these species occur globally. Rodents that pose a threat to human interests are called pest species and can affect food production, damage infrastructure, transmit animal and zoonotic diseases and have a negative impact on biodiversity (Bonnefoy *et al.* 2008; Backhans & Fellström 2012; Capizzi *et al.* 2014; Battersby 2015; Marquez *et al.* 2019).

Rodent control can be performed in different ways (Lund 1975). Both lethal and non-lethal methods are practiced, so when choosing a method pest managers and local authorities have to consider different factors and stakeholders (Baker *et al.* 2016; Carter *et al.* 2020). In addition, laypeople are faced with many options for controlling mice and rat populations in and around their property. The suitability of a method of control depends on the species and the context within which it is used, thereby further complicating the decision (Byers *et al.* 2019; Htwe *et al.* 2019; Pascual *et al.* 2020). Implementing rodent control without first understanding the cause of the pest problem and/or examining the efficacy of methods used, leads to important ecological and economic losses (Stenseth *et al.* 2003; Romero 2016). Another question concerns the impact of pest management on non-target wildlife and ecosystems; therefore, pest control management is often framed within wildlife conservation management debates. One important tool for guiding pest control management is Integrated Pest Management (IPM). This focuses on long-term prevention of pest species using a combination of techniques by minimising pesticide use as much as possible and giving preference to prevention, followed by mechanical and biological techniques (Witmer 2018). This approach focuses on prevention by proactive management, as the aim is to minimise the use of chemical methods. Ecologically based Rodent Management further widens this approach by explicitly considering underlying ecological mechanisms leading to problematic rodent populations (Singleton *et al.* 1999).

Nowadays, rodents are recognised as sentient animals and humane management is preferred (Broom 2014; Hampton *et al.* 2020). Wolf and Schaffner (2019) describe a shift in pest management ethics over time, whereby the recognition of the intrinsic value of non-human animals and our moral obligation to treat them with compassion is receiving increasing attention. Several researchers have described the rise in animal welfare concerns as something which can no longer be ignored in pest management control methods (Hadidian 2012; Dubois *et al.* 2017; Gilhofer *et al.* 2019; Davey & Zhao 2020; Wolf & Hamilton 2020). Baker *et al.* (2020) report that users wanted pest control methods to be quick, sustainable, and safe for people and non-target species while, with regard to lethal control methods, the respondents were concerned for animal welfare. In addition, pest controllers themselves have ethical or emotional objections to poorly functioning control methods that cause animal suffering. Van Gerwen *et al.* (2020) found that almost half of the Dutch pest controllers they interviewed find it difficult to weigh animal interests against those of humans. The growing demand for more rodent control methods that minimise suffering makes it necessary to include animal welfare parameters when dealing with mice and rats as pests (Paparella 2006; Beausoleil & Mellor 2015), moreover Yeates (2010) states that pest management can learn from laboratory animal ethics and wildlife management.

Until now, much of the research on the impact of rodent control methods has focused on the effectiveness with which the population was reduced, on impact on humans and wildlife, or environmental impact (López-Perea & Mateo 2018; Fischer *et al.* 2019). Unlike for mice and rats kept as laboratory or companion animals, very little attention is paid to the welfare of these species as a pest. For example, European and Flemish legislation on animal welfare is based on humans’ relationship with other species such as agricultural animals, laboratory animals, companion animals, zoo animals, circus animals and so on (Regering 2020). Protection of welfare is included in concurrent legislation and protocols (Hurst & West 2010; Spangenberg & Keeling 2015; Hawkins *et al.* 2016; Lidster *et al.* 2016; Prescott & Lidster 2017). According to Smit (2015) the concept of integrated pest management (IPM) is currently lacking certain elements which would enable full implementation of humane pest management strategies. Various stakeholders have called for humane pest management and sound research, delivering accurate and reliable information and good practices (Sharp & Saunders 2004; Hampton *et al.* 2016; van Gerwen *et al.* 2020). Although the pest management industry and pest control regulatory bodies have already developed codes of practice attempting to integrate animal welfare, and although science already provides frameworks to assess the animal suffering caused by pest control methods (eg Sharp & Saunders 2011), scientific knowledge on this topic needs to be explored and expanded (Sharp & Saunders 2004; Universities Federation for Animal Welfare [UFAW] 2006, 2009; Talling & Inglis 2009; Australian Environmental Pest Managers’ Association [AEPMA] 2019).

This study investigates the degree of suffering endured by the target rodents for different control and dispatch methods. Since empirical data about the direct impact of these methods are limited, we opted to collect expert opinions and analyse these data using a systematic scoring method. The outcome of our study can help both laypeople and professionals making well-informed decisions about how to control mouse and rat populations and can also guide regulatory agencies and legislators in developing policies and legislation.

## Materials and methods

For the purpose of this study, a range of legal methods for the control of mice and rats were selected. A panel of experts was compiled to score animal welfare parameters and an expert focus group session was organised in order to arrive at consensus scores.

### Selection of common control and dispatch methods

The control methods were selected based on expert judgement and with the criterion that they had to be legal for use in Belgium (ie on the list of approved biocides) (FOD 2020). As a result of the plethora of available methods, fourteen lethal and non-lethal methods to control or dispatch rats and mice were chosen for evaluation. The two non-lethal methods were live capture traps, i.e. containers that allow one or more animals to enter but not exit and glue board traps, i.e. cardboard boards covered with a strong adhesive. Both methods can lead to death when used inappropriately. During the scoring process, it became clear that single-capture and multiple-capture traps needed to be treated separately (see *Results*). The most common methods used to dispatch trapped animals include drowning, deprivation (i.e. holding captured animals so long that they die due to stress, or lack of water, food, or warmth) and cervical dislocation. Trap and relocation of species was discussed in the preparatory report but not included for the experts since it is not legal to do so in Belgium. For lethal control methods in the category of mechanical killing traps, snap traps (also known as break-back traps), electrocution traps and captive-bolt traps were selected. The captive-bolt trap is not yet in common use but has been approved and is advertised as very humane. Snap traps are probably the best known traps and work by forcefully hitting and then grasping the animal’s back, neck or skull with a spring-loaded metal or plastic bar or closing jaw. Electrocution traps are small boxes with two metal plates; once an animal steps onto these plates an electric current is sent through its body leading to death. The captive-bolt trap works by rapidly expelling a metal rod which hits the animal in the head and crushes its skull resulting in death. For chemical control agents, all substances legal for use in Belgium were scored. These include anticoagulant rodenticides (vitamin K antagonists), which interfere with the blood clotting cycle resulting in death after a few days due to internal haemorrhages. Aluminium phosphide works by releasing phosphine gas, which forms when the aluminium phosphide comes into contact with humidity in the environment. This gas causes death to the species in sufficiently high concentration. Chloralose works by diminishing brain activity causing hypothermia and eventually death (when the ambient temperature is below 16°C). Carbon dioxide is used in reduced-sized traps that release a small amount of the gas whenever an animal enters the trap, and the gas inhibits the uptake of oxygen. The same is true for hydrogen cyanide, a fumigant that is used in large-scale applications (i.e. fumigation of entire buildings). Both carbon dioxide and hydrogen cyanide cause death within a few minutes. Lastly, cholecalciferol, which recently came onto the Belgian market, causes calcification of the internal tissues leading to renal and heart failure and death within a few days. We have to acknowledge that some of the chosen methods are illegal in various other countries, e.g. drowning as a dispatch method and glue board traps.

### Scoring system of animal welfare per control method

We used an existing and validated model by Sharp and Saunders (2011), intended to evaluate the effect of a population control method on individual animals and to compare the welfare impact of the different methods. The Sharp and Saunders model uses the Five Domains model of animal welfare (Mellor & Reid 1994) as a basis for the development of a system to assess the effect of experiments, teaching and testing procedures on animals.

The model has two parts (A and B) for assessing welfare impact. Part A examines the ‘impact on the animal’ and the ‘duration of the suffering caused’ of a non-lethal method or the preceding, non-lethal stage of a lethal method. First, animal impact scores (no impact, mild impact, moderate impact, severe impact or extreme impact) are attributed for five domains: (1) water deprivation, food deprivation or malnutrition; (2) environmental challenge; (3) injury, disease, functional impairment; (4) behavioural, interactive restriction; (5) anxiety, fear, pain, distress, thirst, hunger. Second, the duration of the impact (immediate/seconds, minutes, hours, days, weeks) is determined. Based on both impact attributions, the overall Welfare Impact (WI) is calculated, ranging from 1 (no impact) to 8 (maximum impact) for increasing levels of suffering. For the lethal control and dispatch methods, part B is added to the score and incorporates the ‘actual mode of death’ and the ‘duration of the induced suffering to unconsciousness.’ First, scores are attributed to the level of suffering (no suffering, mild suffering, moderate suffering, severe suffering or extreme suffering). Second, the time until unconsciousness is determined (immediate/seconds, minutes, hours, days, weeks). The final score for part B is defined on a scale from A (no impact) to H (severe impact) for increasing levels of suffering, which is called the Death Impact (DI). Assessment of the welfare impact of a non-lethal method is thus based on part A (score range 1–8) only, whereas for a lethal method parts A and B are combined (score range 1A–8H).

### Panel of experts and focus group session for the scoring of the animal welfare parameters

One of the important prerequisites of the model is that welfare assessments should be conducted by a panel of experts (Sharp & Saunders 2011; Baker *et al.* 2016). Participants were invited according to a pre-determined priority based on expertise. Eight experts agreed to participate in the study. The panel included: a practicing veterinarian who specialised in rodents, two professional pest control officers, one toxicologist, a laboratory animal science expert, a pest control researcher, an animal population modeller who specialised in rodents and an animal welfare expert.

Expert assessments have to be based on *“*the proper implementation of the control methods as set out in the standard operating procedures and impacts should be predicted as much as possible on the basis of a review of the relevant human and animal literature, so that an appropriate sampling regime can be set up*”* as stated by Littin and O’Connor (2002). Consequently, even though the participating experts were selected based on their field of expertise and expected to be familiar with the existing literature, a background reference document with useful information (biological characteristics and control methods) was compiled and made available to the experts. This document contained a comprehensive summary of the biological features of the three target species (house mouse, Norway and black rat), and information about the standard operating procedures that included model examples for each control or dispatch method and its mode of action.

Both the background reference document and the scoring form were piloted with five researchers in order to evaluate comprehensibility and ease of use. This resulted in some re-wording to ensure clarity and a methodological adjustment was made to the instructions as originally outlined by Sharp and Saunders (2011).

**Figure 1.**
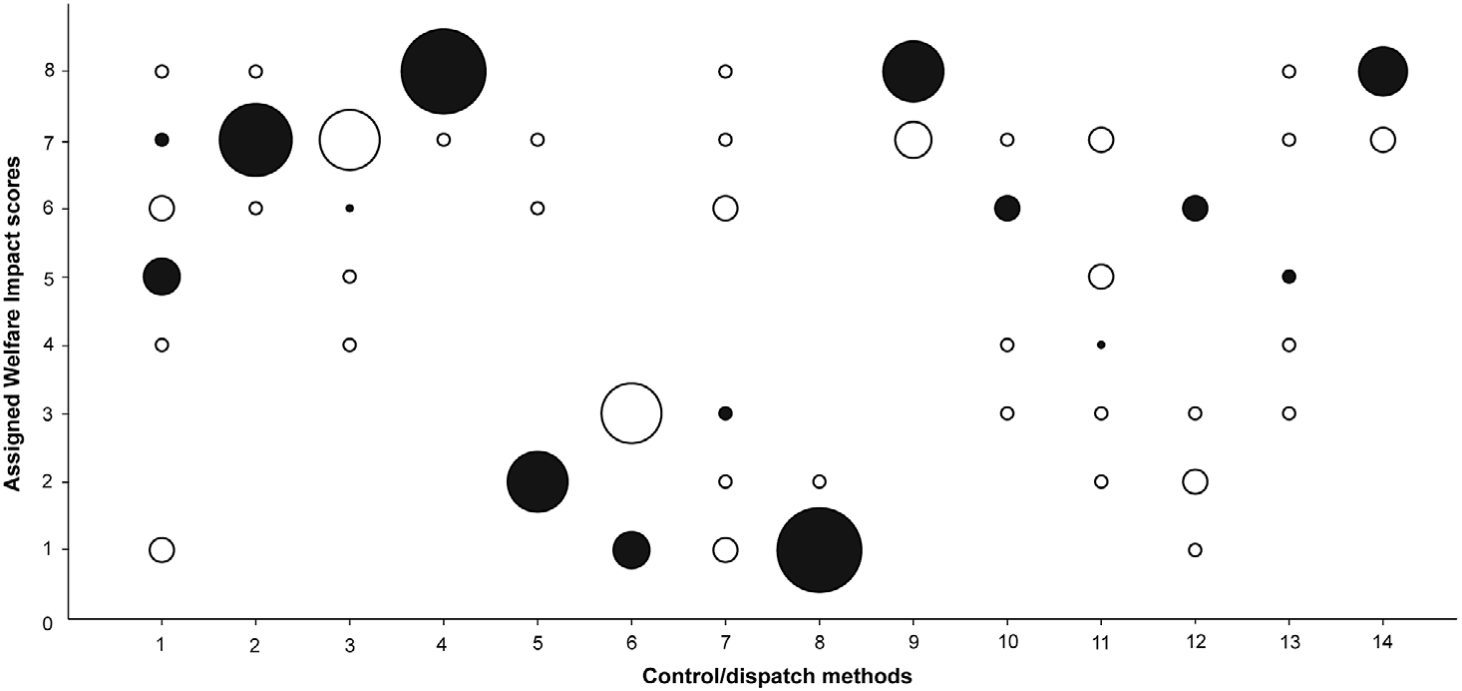
*Frequency table of Welfare Impact scores (1 through 8) assigned by individual experts prior to the focus group discussion for each of the control/dispatch methods (Part A).* Control/dispatch methods: (1) live capture trap, (2) glue board trap, (3) drowning, (4) deprivation, (5) cervical dislocation, (6) electrocution trap, (7) snap trap, (8) captive-bolt trap, (9) anticoagulants, (10) aluminium phosphide, (11) chloralose, (12) carbon dioxide, (13) hydrogen cyanide and (14) cholecalciferol. The size of the circles indicates the number of experts that gave that score. The black-filled circles indicate the final consensus scores. The live capture trap has two final consensus scores, due to split up after focus group discussion: LCT1: live capture trap 1 animal, consensus score 5; LCT+: live capture trap multiple animals, consensus score: 7.

**Figure 2.**
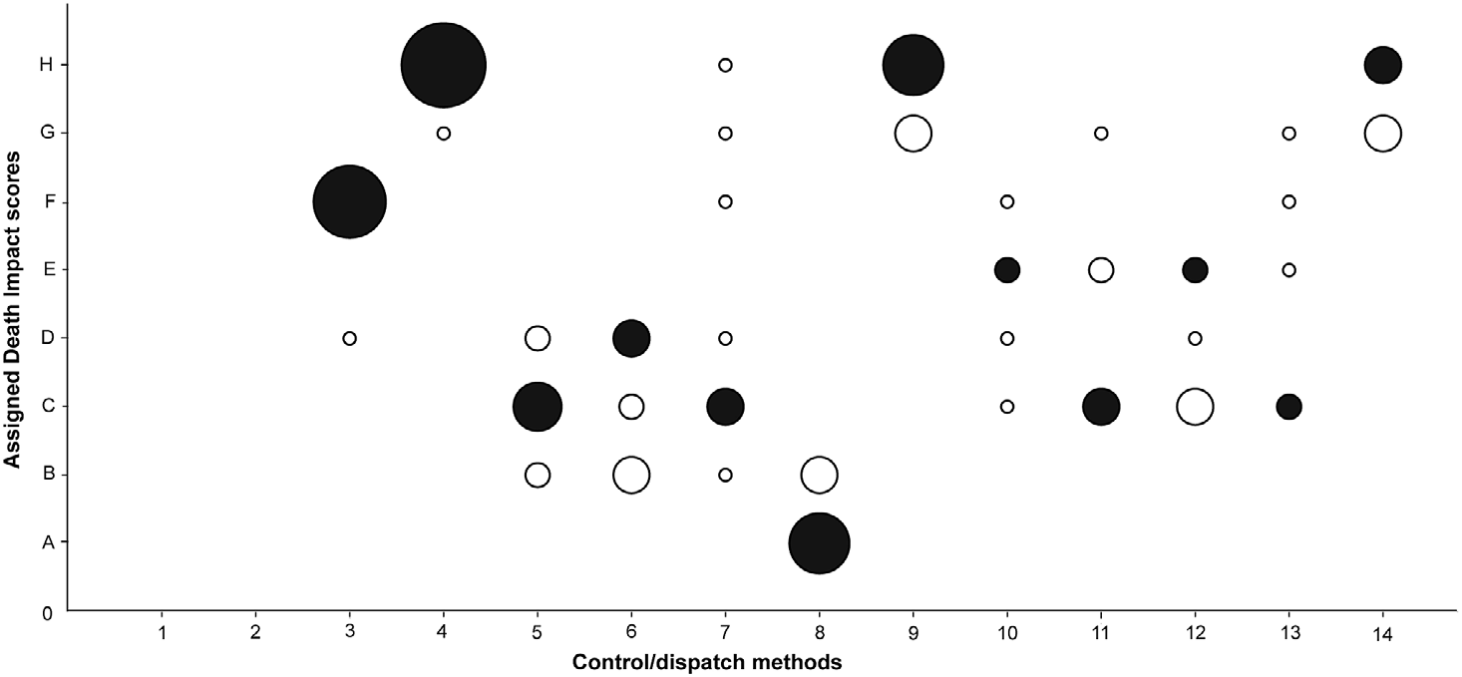
*Assigned Death Impact scores (A through H) by individual experts prior to the focus group discussion for each of the control/dispatch methods (Part B).* Control/dispatch methods: (1) live capture trap, (2) glue board trap, (3) drowning, (4) deprivation, (5) cervical dislocation, (6) electrocution trap, (7) snap trap, (8) captive-bolt trap, (9) anticoagulants, (10) aluminium phosphide, (11) chloralose, (12) carbon dioxide, (13) hydrogen cyanide and (14) cholecalciferol. The size of the circles indicates the number of experts that gave that score. The black-filled circles indicate the final consensus scores.

Panel members received the background reference document and the score-sheet with instructions. The experts were asked to submit their individual scores and justifications to the focus group leader prior to the focus group discussion. Experts were asked to give a general score for the three rodent species combined, but if there were reasons to expect large differences between the three species the expert was expected to point this out in his or her scoring sheet. A methodological adjustment for scoring was made, in line with the findings of “over and underestimation of impacts” in the paper by Baker *et al.* (2016). More specifically, this draws on the principle for use: “Where there is doubt or lack of objective knowledge about whether an animal will suffer severely, one should assume it will do so, i.e. the ‘benefit of the doubt’ should be given in favour of the animal.” We dropped this principle and gave the experts the opportunity not to score the impact in their individual scoring phase, with the purpose of further exploration in the focus group discussion. It is methodologically important to have the possibility of not scoring a method and instead having an exchange of views on it. Otherwise, if panel members are forced to make best guesses this can lead to assessing certain methods as less or more harmful which, in the end, can lead them to be less critical of the use of methods. This adjustment resulted in two methods receiving five initial individual expert scores (aluminium phosphide and hydrogen cyanide), three methods receiving six initial individual expert scores (chloralose, carbon dioxide and cholecalciferol) and two receiving seven initial individual expert scores (drowning and cervical dislocation). All the other methods were individually scored by all eight experts (Figures 1 and 2).

The individual scores were summarised and an online focus group session with the experts was organised in order to arrive at a final consensus score for each method. The goal was to achieve consensus on the divergent scores by clarifying the underlying reasoning of the experts and to have a final substantiated score for all methods. This session was organised as follows: First, the scores for which there was a clear consensus were approved by the experts. In a second extensive phase, scores for which there was a lot of variation needed to be discussed in order to be agreed upon. In a third phase, the scores for all methods were examined as a whole. After the focus group session, the experts received a report with consensus scores and argumentation for final approval and comments, and a final report was delivered.

## Results

### Individual and consensus scores

**Figure 3.**
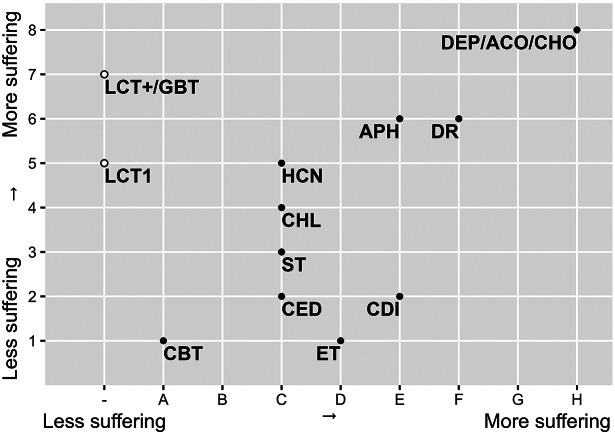
*Graphical presentation (after Sharp & Saunders* *2011* *) of the expert panel consensus on the animal welfare scores of the fourteen population control methods for mice and rats.* The x-axis indicates the time until unconsciousness and degree of suffering for lethal methods (part B); the y-axis indicates a method’s impact on welfare before death (non-lethal phase) (part A). LCT1: live capture trap 1 animal; LCT+: live capture trap multiple animals; GBT: glue board trap; DR: drowning; DEP: deprivation; CED: cervical dislocation; ET: electrocution trap; ST: snap trap, Captive-bolt trap: CBT; ACO: anticoagulants; APH: aluminium phosphide; CHL: chloralose; CDI: carbon dioxide; HCN: hydrogen cyanide and CHO: cholecalciferol.

The results of both the Welfare Impact scores (Figure 1) and the Death Impact scores (Figure 2) show clearly differentiated scores for the different control and dispatch methods. Multiple capture live traps are more likely to result in suffering than single live traps. Also, Glue board traps received a high score. Among the dispatch methods, drowning and, even more so, deprivation cause considerable suffering, while cervical dislocation is considered to have a smaller impact. Electrocution traps, snap traps and captive-bolt traps are thought to cause less suffering during the capture process, but electrocution likely causes more suffering than the other methods during the killing itself. The different chemical methods have intermediate scores, except for anticoagulants and cholecalciferol which both are considered to have a strong impact on animal welfare and result in death with a lot of suffering (Figure 3).

### Additional clarification for certain scores

Based on their discussions in the focus group, the experts added additional clarifications for the scores for eight of the control methods. For the live capture trap, the experts recommended making a distinction between traps that are built to catch a single animal and those that can catch multiple animals (a score of 5 for traps for one animal and a score of 7 for traps for multiple animals). With the latter, the risk of physical fighting and fighting-related injuries between multiple individuals of the same species can influence the animal welfare impact, as can being trapped simultaneously with non-target species (Belant & Windels 2007; The British Columbia Society for the Prevention of Cruelty to Animals 2019; Waudby *et al.* 2019).

The glue board trap was given an impact score of 7, but the experts added that a score of 8 would be more appropriate if the traps are not checked several times a day (Kim *et al.* 2007; Fenwick 2013). The drowning impact score of 6F was attributed on the condition that drowning is understood as direct immersion in water and not swimming until exhaustion (Bierens *et al.* 2016; Hyodoh *et al.* 2016). The experts commented for Norway rats, in particular, since swimming is part of their normal behavioural repertoire, it is important they should be immersed immediately (Kramer *et al.* 1993).

The experts decided to give the snap trap a consensus score of 3C. They pointed out, however, that this score is conditional upon correct placement and good quality of the snap trap, otherwise the score could be considerably higher. Experts indicated there to be great variation in types of snap traps as stated by Baker *et al.* (2012). Many things can go wrong with this method because laypeople often place snap traps incorrectly or use low quality traps that may not kill the trapped animal immediately (Pawlina & Proulx 1999; German Environment Agency 2020). This is also the case when, for example, mouse snap traps are used for catching rats. In addition, trapping non-target species could be a problem, especially in an outdoor situation.

The experts assigned a score of 1A to the captive-bolt trap but stressed that, given the novelty of this trap, more information about optimal placement is still needed (Walsh *et al.* 2017; Shiels *et al.* 2019; Bogardus & Shiels 2020; McRae *et al.* 2020).

After deliberation, the experts assigned aluminium phosphide a score of 6E. In particular, the dose and duration were matter of debate (Snider 1983; EFSA 2009). Among other things, the slow release of gas and the very strong convulsions evoked determined the score (Meehan 1984; Anand *et al.* 2012; Shakeri & Mehrpour 2015; Tahergorabi *et al.* 2020).

Chloralose was attributed a score of 4C. The score was given, among other considerations, due to the excitation at the induction during the first phase (Austin *et al.* 2005; Segev *et al.* 2006; Pelfrène 2010). The experts expressed concerns about dose intake of chloralose (Federal Public Service Health 2019; Pepin *et al.* 2020). In addition, they noted that rats may not eat a sufficient amount, in contrast to mice which tend to do so.

The initial individual scores for hydrogen cyanide varied widely. The main reason was the difficulty in interpreting how the gas was released based on the description provided. The time-span of release plays a major role in assessing the impact of this method (Sousa *et al.* 2003; DeLeon *et al.* 2018; Rice *et al.* 2018). The experts concluded with a score of 5C within the limits of interpreting the procedure for this method.

## Discussion

Combining the assessment of part A (the impact on animal welfare during the period the animals are still alive) and part B (the impact of the killing itself) allows identification of the control methods’ overall effect on the suffering of the target animal. The combined results indicated that, according to the expert judgement, the captive-bolt trap and the electrocution trap cause least suffering, with decent snap traps or single-capture live traps combined with cervical dislocation to dispatch the animal, as adequate alternatives. The use of anticoagulants or cholecalciferol was judged to cause considerable suffering.

Considering the results from lowest score to highest, our results resonate with the findings of Fisher *et al.* (2016, 2019), Sharp and Saunders (2011) and Mason and Littin (2003). These results are also in line with a similar study that very recently assessed animal welfare impacts in the management of Norway rats (Baker *et al.* 2022). Our experts gave a low suffering score for the captive-bolt trap, with the caveat that careful placement seems crucial for getting animals into the trap (Van Horne 1982; Walsh *et al.* 2017; Shiels *et al.* 2019; Bogardus & Shiels 2020; McRae *et al.* 2020). Shiels *et al.* (2019) split up their assessment for the effect of the captive-bolt trap on rats and mice, suggesting that the captive-bolt trap would not be suitable for trapping mice. Our high suffering score for glue board traps is in accordance with the findings of Fenwick (2013), who pointed out that the use of glue traps for rodent pest control does not meet established standards for humane restraint. Concerning the use of drowning as dispatch method and the use of glue traps, the experts were clear that these methods are considered inhumane. The harm done to the animals when using one of these techniques is obvious and their use is already banned or restricted in several countries (e.g. France, United Kingdom, Sweden, The Netherlands). Also, the use of anticoagulant rodenticides is increasingly restricted, although mainly for environmental reasons. This leads to the striking finding that this method, with the worst impact on animal welfare, is representative of the majority of the market. So anticoagulant rodenticides seem to legally slip through the cracks. It is clear that sensitisation of the general public is needed to overcome this misconception. In this way the real impact of anticoagulant rodenticides on animal welfare is revealed, which can influence the public opinion. Depending on correct functioning and placement, as assessed by Baker *et al.* (2012), snap traps can be considered as more humane than rodenticides. Our findings confirm those of Nattrass *et al.* (2019), Schmolz and Friesen (2020) and the German Environment Agency (2020) whereby live traps tend to be a more humane way of killing than rodenticides, as long as they are frequently checked and followed by a suitable killing method such as cervical dislocation. In accordance with Flammer *et al.* (2019) and Steiner *et al.* (2019), our expert panel’s assessment suggests that carbon dioxide is not the best option, as the gas is aversive to rodents and exposure to it while animals are conscious may be inhumane. Although the effect of carbon dioxide can vary based on the inhaled concentration, its use as a rodenticide is not advised.

The experts also indicated that the standard instructions supplied with the methods should contain more details on proper implementation since traps are often mishandled. Based on species-specific features, experts also insisted on drawing attention to various risks such as impact of weather conditions and direct exposure to, for example, precipitation, bright sunshine, presence of predators, distress/panic due to other environmental stimuli, an animal’s condition (e.g. thirst/health, injuries due to capture or escape attempts), duration of confinement, access to food/water and time needed to remove the animal from the trap during control (Berry *et al.* 1984; Drickamer & Paine 1992; Castelhano-Carlos & Baumans 2009; Crowcroft & Jeffers 2009; Fullerton Hanson & Bardoy 2010; Tynes 2019).

The Sharp and Saunders model proved to be a sound approach in terms of scoring. One adjustment was made in the individual scoring instructions on attributing scores where information was missing or uncertainty arose, and this strengthened the experts’ deliberation process. In addition, the delineation of both parts A and B could be further refined for use.

During the group session, the interpretation of the scoring method of both parts was aligned, as there was often overlap in the duration of both. The experts indicated several times that the division into two parts sometimes made lethal method scoring difficult. This may also explain why anticoagulants received a score of 8 for part A in the present study but a score of 1 in the Sharp and Saunders study (2011). Sharp and Saunders (2011) also indicate in their model description why they awarded this score on page 41, “With methods involving toxic baits it is likely that there will be no welfare impact prior to the animal ingesting the bait, therefore it is not necessary to assess both part A and B. Only part B is required.” In the recent Baker *et al.* (2022) study, anticoagulants were given an intermediate score for part A based on the mild impact during most of the period of deployment. It should be noted a study was carried out to reduce the pain of anticoagulants (Food and Environment Research Agency 2011). This is an example of how the suffering impact of anticoagulants can be improved, but further research is needed.

There was a wide diversity of individual expert scores for several methods, both in the individual Welfare Impact scores (Figure 1) and the Death Impact scores (Figure 2). During the focus group session, two main reasons for these diverging scores became clear. On the one hand, the lack of details in some of the standard operating procedures described in the background document lead to different interpretations of the impact of methods; on the other, the experts’ understanding of what constituted part A and B of the model sometimes differed.

As set out in the ‘Principles for use’ from Sharp and Saunders (2011), the instructions as provided by the manufacturer for the control methods were used as a basis for assessment. However, certain standard operating procedures were open to interpretation and therefore could be made more detailed. For example, snap traps had incomplete instructions, those for hydrogen cyanide were not clear enough and live capture traps had ambiguous instructions. This led to discussions among the focus group experts on the impact of the control method. The experts found that clear, quality standard operating procedures are essential for humane pest control methods, and this was echoed by AEPMA (2019). Proulx *et al.* (2020) also mentioned there are issues with ‘Protocols for the Use of Certified Traps.’ The lack of testing of standard operating procedures poses a threat to humane pest management (Virgós *et al.* 2016; Eason *et al.* 2018) and training is needed to correctly execute certain methods, e.g. cervical dislocation (Martin *et al.* 2018). The inclusion of animal welfare scores in standard operating procedures can be useful for the certification of control methods.

Moreover, it is also important to underline the quality of the material that is commercially available. The occasionally large variation in the quality of a particular control method is highly relevant (e.g. a good snap trap with a larger opening angle and ‘double-peg’ spring mechanism positively affecting momentum and clamping force probably causes much less suffering than a trap of poor quality) (Baker *et al.* 2012). However, a broad range of different quality devices is available on the market and widely used. The Welfare Impact (WI) score varied greatly in the initial individual scoring phase depending on which trap one had in mind. Therefore, the experts welcome the European certification system for mechanical trap types elaborated in the Non-Chemical Alternatives for Rodent Control (NoCheRo) project (ISO 1999; German Environment Agency 2020). In the current proposal, the condition of a maximum duration of 30 seconds before an animal loses consciousness (resulting in death) has to be met for an A-label, while a maximum duration of 180 seconds before loss of consciousness is accepted for a B-label. Devices exceeding this limit are not certified. The phasing out of B labels will be introduced and this is also welcomed by the experts.

The perception that mechanical handling and killing devices are less humane in comparison to poisons is disturbing (Nattrass *et al.* 2019). This underlines the statement of Beausoleil and Mellor (2015) that “Evaluation of the welfare impacts of pest control tools is required to inform the decisions of pest control operators and policy-makers, and to address growing negative public perceptions regarding some methods of pest control.” Schmolz and Friesen (2020) describe a double standard in the assessment of animal welfare of traps and rodenticides, whereby the vague evaluation of rodenticides reinforces the perception that traps are less humane than rodenticides. As the German Environment Agency (2020) states, objective assessment based on criteria such as time until an animal’s irreversible unconsciousness (TIU) would clearly show the opposite in the vast majority of cases. Our results support this statement. This study did not address the question of how TIU can be derived from behaviour and appearance; the determination of TIU, especially on the basis of behaviour and appearance, requires further research.

In addition, IPM would benefit from including animal welfare scores to reduce animal pain, suffering and distress as reported by McMahon *et al.* (2012) and Hampton *et al.* (2019) and efforts should be made to inform the general public and policy-makers about the animal welfare impact of rodent control methods and to promote uptake of the more humane methods (Flor & Singleton 2011).

It is important to keep in mind that the scores attributed only relate to the welfare impact of the method, without taking into account other aspects such as potential variation in the methods’ efficacy. A method may score well from a welfare point of view, yet rodent control managers might experience that method as being somewhat inefficient, lacking selectivity or being unsafe. In addition, the importance of effectiveness is important from an animal welfare perspective. With an effective method, fewer animals will ultimately need to be controlled than with a less effective method whereby a surplus population is always removed, resulting in ‘sustainable killing’ rather than a population decline.

During the focus group discussion, the experts mentioned four points that ought to be emphasised despite being beyond the scope of this study: (i) mouse and rat management ought primarily to be a project of prevention, as stated by IPM; (ii) the risk of implicating non-target species; (iii) the risk of not being able to use the method properly; and (iv) the importance of taking into account effectiveness of the method to reduce a population are important considerations when choosing a control method. It is important to consider how impact on non-target animals can be avoided as much as possible (Virgós *et al.* 2016; López-Perea & Mateo 2018; Proulx *et al.* 2020; Rattner & Harvey 2020).

### Animal welfare implications

The animal welfare impact scoring in this paper of fourteen rodent control and dispatch methods can serve as a guide for those who wish to select the most humane methods for mouse and rat population control, either for laypeople or professionals, or for any policy-making body seeking to regulate or advise on such methods. Proactive management based on preventive measures such as reducing the supply of feed and shelter/nesting areas, should always be recommended. In that case, rats and mice will not be attracted and rodents or populations of rodents will neither thrive nor expand (Buckle & Smith 2015). If there is still a need to control rodent overpopulation, the animal welfare impact should be considered when choosing a method. For this, the consensus expert scores from the current study can be used. Moreover, these indicate that certain commonly used methods, such as anticoagulants, are better discouraged from an animal welfare perspective. Of course, other aspects of the methods, such as effectiveness, species-specificity, labour requirements, safety issues and cost also need to be considered.

Furthermore, research is needed on the animal welfare impact on the three species of the control methods which use hydrogen cyanide, chloralose and aluminium phosphide. In order to make a full assessment of the animal welfare impact, account must also be taken of the effectiveness and of how these methods are used in reality, including the risk of secondary catches.

## Conclusion

We established animal welfare scores for fourteen pest control and dispatch methods, and clear different impacts were identified. From an animal welfare perspective, according to our scores at present, the captive-bolt trap, the electrocution trap, good quality snap traps and single-capture live traps with cervical dislocation as dispatch method are preferable while the use of anticoagulants, cholecalciferol and live trapping combined with deprivation are to be avoided. It should be noted that although the captive-bolt trap achieves the best score, the electrocution trap scores overall similar to good snap traps and single-capture life traps with cervical dislocation as a dispatch method. It should however be noted that cervical dislocation must be carried out properly, which is not possible if a lot of animals have to be killed at the same time by an individual person.

Furthermore, our study underlines the necessity for empirical research on the impact of rodent control methods on animal welfare and for testing of standard operating procedures. Our research also underlined the importance of legal provisions concerning the sale of quality traps and public understanding of the animal welfare impact of different rodent control methods.
